# The Impact of COVID-19 on Burns: A Brazilian Study

**DOI:** 10.3390/ebj4010001

**Published:** 2022-12-28

**Authors:** Carolina Moura, Marcela Bittencourt, Maria Luíza Cazumbá, Alexandra Buda, Alexis Bowder, Daniel Scott Corlew, Fábio Mendes Botelho Filho, Lucas Barboza, Laura Pompermaier

**Affiliations:** 1 Department of Neurology, Universidade Federal Fluminense, Niteroi 24020-140, Brazil; 2 Department of Medicine, Universidade José Rosário do Vellano, Belo Horizonte 31710-030, Brazil; 3 Department of Medicine, Faculdade de Medicina da Universidade Federal de Minas Gerais, Belo Horizonte 31270-901, Brazil; 4 Program in Global Surgery and Social Change—PGSSC, Boston, MA 02115, USA; 5 Department of Hand Surgery, Plastic Surgery and Burns, Linköping University, 581 83 Linköping, Sweden; 6 Department of Biomedical and Clinical Scienes, Linköping University, 581 83 Linköping, Sweden

**Keywords:** burns, epidemiology, COVID-19, Brazil

## Abstract

During the COVID-19 pandemic, some of the strategies chosen to contain the spread, such as social isolation and use of alcohol-based hand sanitizer, were suspected to increase the risk of domestic accidents, especially burns. The aim of this study was, therefore, to investigate possible differences in epidemiological trends among burned patients admitted to the main referral hospital of the State of Minas Gerais, Brazil, before and during the pandemic. Methods: All categories of new burns admitted at the Burn Unit of the João XXIII Hospital in Belo Horizonte, Minas Gerais. The study group consisted of burn patients admitted between 1 March and 31 December 2020, and the control group consisted of those admitted between 1 March and 31 December 2019. The population was analyzed descriptively, and differences between patients admitted before and during the pandemic were tested using t-test, Wilcoxon Mann–Whitney Rank Sum test, the Chi-Squared test or Fisher’s exact test, as appropriate. Results: During the study period, 914 patients were admitted at the burns unit, 535 before the pandemic (control group) and 379 during the pandemic (study group). During the pandemic, referral from other hospitals decreased, while time between injury and admission remained unchanged. TBSA% and LOS diminished, while the depth of burns, presence of inhalation injuries, and in-hospital mortality did not. In adults, the place and mechanism of injury changed during the pandemic, while in children they did not. Conclusion: Fewer patients with burns were referred for specialized burn care during the pandemic, although patients admitted for specialized burn care had smaller TBSA% and shorter LOS.

## 1. Introduction

The rapid spread of the COVID-19 virus led to exceptional strategies which were adopted worldwide to limit the outbreak. Businesses considered to be non-essential were canceled, outdoor activities were limited, and social isolation was recommended [1]. The restrictions resulted in people mostly staying at home, leading to a potential increase in their exposure to domestic accidents, such as burn injuries [2]. In the state of Minas Gerais, Brazil, the first case of COVID-19 was confirmed on 8 March 2020, and the state of quarantine was declared on 18 March 2020. Furthermore, to reduce the spread of the virus, the authorities suggested the use of alcohol-based disinfectants, possibly increasing the risk of flame injuries [1,2]. However, while the rise of burn injuries during the outbreak was anecdotally reported by Brazilian physicians, but not verified [2,3], the increment of flame injuries caused by the external use of hand sanitizer, particularly affecting the arms and the trunk, was recently reported in Minas Gerais [4], suggesting that variations in epidemiological patterns were caused by changed behaviors. Understanding the impact of exceptional events, such as a pandemic, on burn injuries trends is indispensable in order to develop appropriate preventive strategies and to respond to a possible surge. However, it remains to be investigated whether the occurrence, cause, and severity of burns changed during the outbreak. For this reason, this study aimed to analyze possible differences in epidemiological trends in Brazil, comparing patients with burns admitted at the Burn Unit of Belo Horizonte, which is the main referral hospital of the state of Minas Gerais, before and during the pandemic.

## 2. Materials and Methods

### 2.1. Study Setting, Design and Data Collection

This retrospective study took place at the Burn Unit of the Trauma Department of the João XXIII Hospital in Belo Horizonte, Minas Gerais, which is in South-East Brazil and is the second most populated state of the country. The João XXIII Hospital was founded in 1973 and is one of the largest trauma-referral hospitals in Latin America [5,6]. All patients with acute burns admitted to the Burn Unit from 1 March to 31 December 2019 and from 1 March 2020 to 31 December 2020 were included in this study. Minor burns, which did not require highly specialized burn care and were managed directly at the Emergency Department and discharged within 6 hours, were excluded. Patients younger than 18 were defined as pediatric burns. Patients with burns who were admitted several times for different burn injuries were considered as separate cases. Data of interest were collected from medical records from the Burn Unit between January and June 2021. Categorical variables were sex (male/female), age groups (adult burns/pediatric burns), the place where the injury happened (at home, at work, outside), cause of burns (scalds, flame, chemical, electrical, contact burns, other), mechanism of burns (accident, caused by other, self-inflicted, other), referral from other hospitals, location of the burn on the body (head and neck, trunk, arms, hands, genitalia, legs, feet), the presence of full thickness burn, in-patient management, presence of inhalation injury, need for mechanical ventilation, and in-hospital mortality. Continuous variables were age (years), calculated using the difference between admission date and birth date, time to admission (days), calculated using the difference between date of admission and date of burn injury, length of stay at the hospital (LOS, in days), and the percentage of the total body surface area burned (TBSA%).

### 2.2. Statistical Analysis

The study group consisted of burn patients admitted between 1 March and 31 December 2020, while the control group consisted of those admitted between 1 March 2019 and 31 December 2019. Descriptive statistics were calculated with frequency (%) for categorical variables and median (interquartile range, IQR) for continuous. Comparison between groups was analyzed with t-test statistics for normally distributed continuous variables, the Wilcoxon Mann–Whitney Rank Sum test for non-normally distributed continuous or ordinal variables, and Chi-Squared or Fisher’s exact tests for binary or categorical variables. Differences between injuries caused by the external use of alcohol, before and during the pandemic, were analyzed using a Chi-square test. For all analyses, the significance level of 5% was considered. All analyses were conducted in R v4·03 (R Core Team, Vienna, Austria) [7].

## 3. Results

Between 1 March 2019 and 31 December 2019 and from 1 March to 31 December 2020, a total of 781 patients with burns were admitted to the Burn Unit, and 95% of them were managed as in-patients (*n* = 770). Most injuries were accidental (*n* = 603, 86%) and happened at home (*n* = 478, 65%), most patients were adults (median 32 years old), and male sex was predominant (*n* = 474, 60%). The median TBSA% was 15 (IQR 8-28), 40% of patients had full thickness burns (*n* = 312), and 15% had inhalation injuries (*n* = 117).

**Figure 1:**
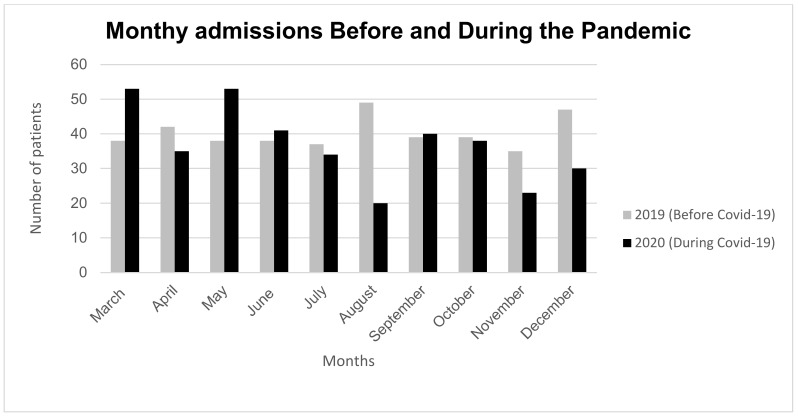
Monthly burn admission before and during COVID-19.

**Figure 2:**
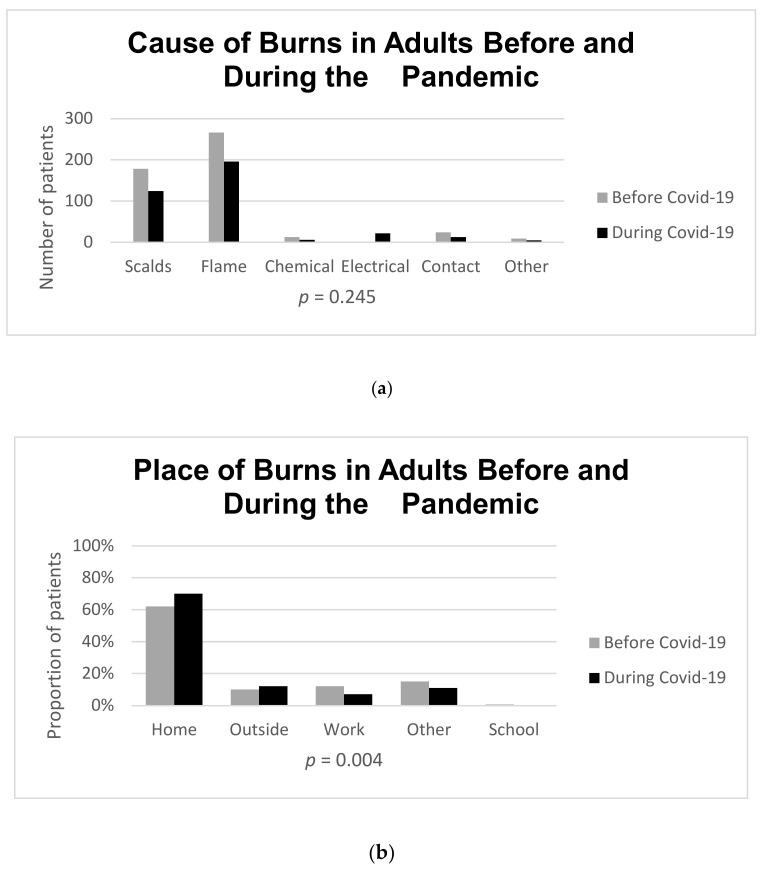

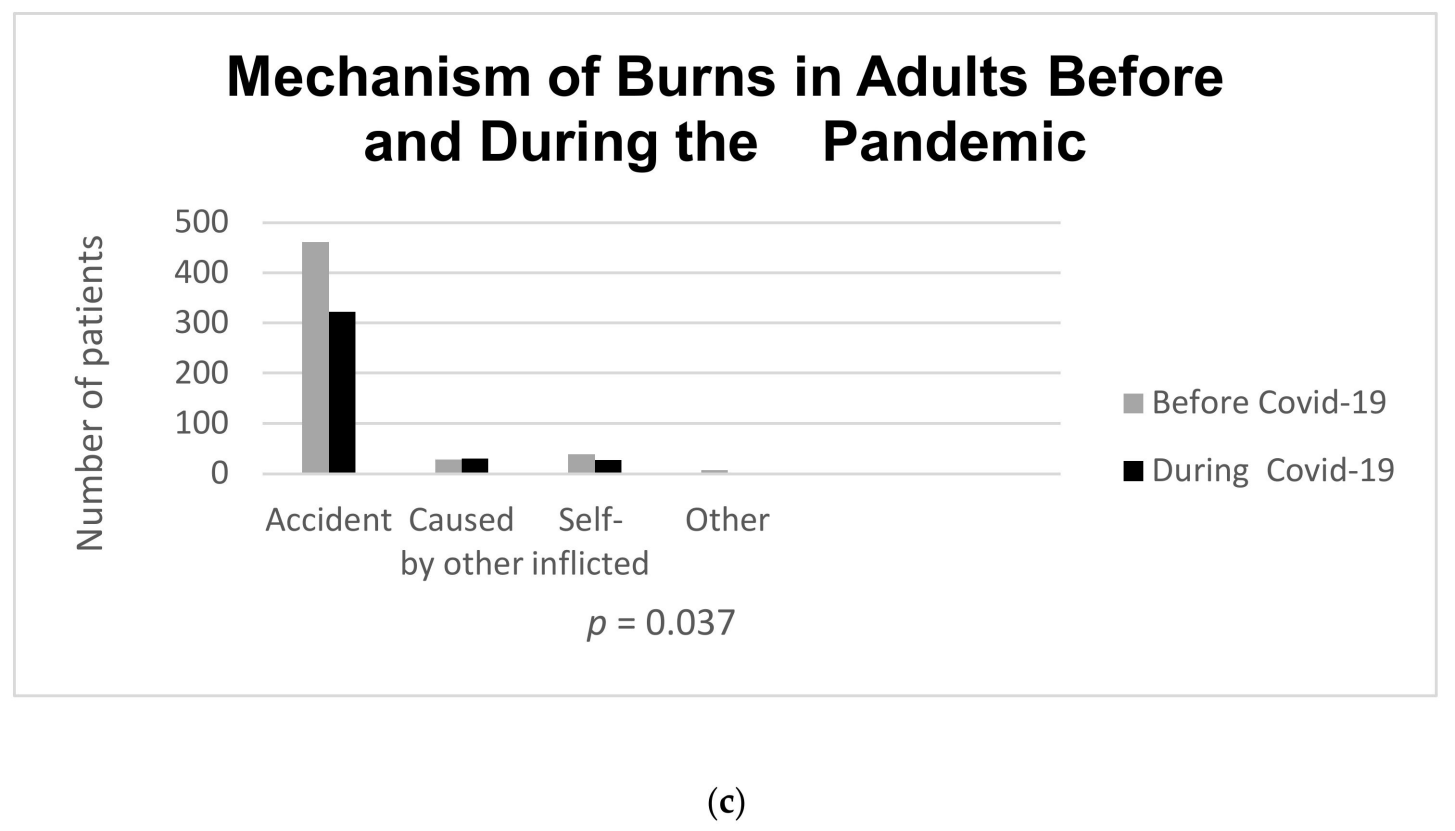
(**a**) Cause of Burns in Adults Before and During the Pandemic: comparison of adult burn patient cause and pandemic (before/during)—Fisher’s exact test, *p*-value = 0.245. (**b**) Mechanism of Burns in Adults Before and During the Pandemic. Mechanism of Burns in Adults, Before and During the Pandemic. Comparison of adult burn patient mechanism and pandemic (before/during)—Fisher’s exact test, *p*-value = 0.037. (**c**) Place of Burns in Adults, Before and During the Pandemic: comparison of adult burn patient place and pandemic (before/during) Chi-squared test, *p*-value = 0.002.

Table 1 shows a comparison between the study and control population. There was a difference in places where injuries happened, with fewer work-related and more domestic accidents during the pandemic compared with those admitted before (*p* < 0.05), whereas the age of patients, sex distribution, and causes of injuries remained unchanged. Referrals from other healthcare facilities to the Burn Unit decreased during the pandemic (*p* < 0.05), and so did the LOS (*p* = 0.014), while the time between injury and admission at the Burn Unit remained unchanged. During the outbreak, TBSA% diminished (*p* = 0.038), while the presence of full-thickness burns and of inhalation injuries did not (*p* = 0.415 and *p* = 0.812, respectively). Monthly burn admissions during the study period are shown in Figure 1. Pediatric burns comprised one-third of all admissions (*n* = 277). The most frequent causes of burns were scalds and flame injuries that occurred accidentally at home, both before and during the pandemic, as shown in Table 2. Among adults, there were differences in the place of injury and mechanism of injury before and during the pandemic (*p* = 0.002 and *p* = 0.037, respectively), whereas the causes of burns remained unchanged (*p* = 0.245), Figure 2a–c. In our study, domestic accidents increased in the study group during the pandemic, whereas work-related injuries remained unchanged (*p* = 0.245), Figure 2a–c.

## 4. Discussion

During the COVID-19 pandemic, the sex and age distribution of patients admitted to the Burn Unit of the João XXIII’s Hospital in Belo Horizonte, Brazil, did not change compared to the pre-pandemic period, confirming reports of other Brazilian studies. [3,4]. The predominance of male sex among burn injuries in South America is 62%, while the predominance of burn injuries in children is around 24%, with no difference in the gender. [3,4,8]. The closure of school and the reduction in outdoor activities imposed during lockdown led to a presumed increase in domestic burns, at least among children, due to exposure to more risk factors, such as playing in the kitchen while cooking or when parents are distracted by teleworking [2,6,9]. Given these premises, a younger median age was expected in patients with burns admitted during the pandemic, but this was not the case, with age remaining the same in study and control group. A plausible explanation of this finding is that, because of adults and children staying at home throughout the day, to carry out household chores, it was necessary for parents to share tasks, such as cooking or guarding children, improving the supervision of children.

However, previous studies had highlighted that burn injuries happen more frequently at home during the colder seasons, when people spend more time indoors, suggesting a seasonality of these kinds of injuries [10,11]. In fact, another study demonstrated that domestic accidents, particularly those happening in the kitchen, increased in the study group during lockdown, whereas work-related injuries decreased [12,13]. Furthermore, other authors showed that flame burns occurred more frequently at home [4], and this study indicated that the incidence of flame burns caused by the external use of alcohol increased during the outbreak. It is probable that the lockdown ensured that people had to cook all of their meals at home using gas-based flames, and this fact, together with the increased use of alcohol-based sanitizers, amplified the risk of domestic flame accidents 185 [14,15,16,17]. An increase in hand burns caused by the external use of alcohol sanitizers was observed in Belo Horizonte, as well as in other Brazilian cities [4]. Even if hands represent only 1% of the TBSA, hand burns require frequently specialized competence for appropriate management, and therefore are listed among injuries that should be referred to the burn unit, according to international guidelines [18,19]. However, during the study period, the location on the body of burns did not change.

During the pandemic, referrals from other healthcare facilities to the Burn Unit in Belo Horizonte decreased, but the retrospective examination of medical records could not explain the reason for this. It has recently been shown that during the outbreak, the fear of contagion prevented people from seeking care [12,13,14,15], which could lead to assumptions that only severe injuries received care. Yet, contrary to what was expected, during the lockdown, burns admitted to the Burn Unit were significantly smaller, while the presence of full thickness burns and of inhalation injuries remained unchanged. One possible explanation is that peripheral hospitals preferred to manage burns locally rather than referring them to the burn center; another is that people were burned less severely. Unfortunately, these hypotheses will remain unsolved without access to data from other hospitals and pre-hospital data. However, the length of stay (LOS) was shorter during the pandemic, which mirrored the well-known relationship between hospitalization time and burn severity, with patients with lower TBSA% requiring less intervention and therefore shorter LOS [14]. Finally, the proportion of patients who were admitted for in-hospital care did not change during the study period, suggesting that the burn unit did not adjust burn management-praxis implementing tele-medicine, as reported by other centers, whereas in-hospital mortality remained unchanged [11,20].

Our study has several limitations. The data were retrieved from medical records, the quality of which depends on the accuracy of the doctors who recorded them. As a consequence, missing data were found in all collected variables, in particular regarding referral status, the place where injury happened, and the presence of inhalation injuries. Data on provided burn care were not collected, preventing us from understanding whether admitted burns required specialized burn care, or the relation between burn severity and LOS.

## 5. Conclusions

Less patients with burns were referred for specialized burn care during the pandemic, although patients admitted for specialized burn care had smaller TBSA% and shorter LOS. The implementation of standardized data collection in dedicated burn registries, including provided medical procedures, might contribute to a better understanding of changes in epidemiological trends.

## Figures and Tables

**Table 1:** Comparison between the study and control population.

	All Patients (*n* = 781)	Control Group (*n* = 402)	Study Group (*n* = 379)	*p*
**Sex**				0.767 ^
Female	307 (40%)	156 (39%)	151 (40%)	
Male	474 (60%)	246 (61%)	228 (60%)	
Age (years) *	32 (9–48)	32.5 (13.75–48)	32 (6.25–48)	0.342 ^#^
**Place of injury**				0.002 ^†^
Outside	81 (11%)	38 (5%)	43 (12%)	
Work	69 (9%)	44 (12%)	25 (7%)	
School	3 (0.4%)	3 (0.4%)	0 (0%)	
Home	478 (65%)	218 (29%)	260 (35%)	
Other	103 (14%)	62 (8%)	41 (5%)	
**Cause of burn**				0.245 ^†^
Scalds	258 (33%)	134 (34%)	124 (33%)	
Flame	397 (51%)	202 (50%)	195 (52%)	
Chemical	23 (3%)	7 (2%)	16 (4%)	
Electrical	56 (7%)	34 (8%)	22 (6%)	
Contact	31 (4%)	18 (4%)	13 (4%)	
Other	10 (2%)	5 (2%)	5 (1%)	
**Mechanism of burn**				0.037 ^†^
Accident	603 (86%)	341 (86%)	322 (85%)	
Caused by other	53 (6%)	23 (5%)	30 (8%)	
Self-inflicted	58 (7%)	31 (7%)	27 (7%)	
Other	7 (1%)	7 (1%)	0	
**Transferred from another city**	371 (46%)	191 (50%)	147 (40%)	0.003 ^
**Time to admission the at the João XXIII**				
Hospital (days) *	1 (0–4)	1 (0–4)	1 (0–3.5)	0.828 ^#^
TBSA% *	15 (8–28)	15 (9–30)	13.5 (7–25)	0.038 ^#^
**Location of the Burn**				0.928 ^
Head and neck	470 (51%) 571 (62%)	284 (53%) 345 (64%)	186 (49%) 226 (60%)	
**Core (Thorax, Abdomen, and Back)**				
Arms	577 (63%)	345 (64%)	232 (61%)	
Hands	188 (21%)	96 (18%)	92 (24%)	
Genitalia	86 (9%)	50 (9%)	36 (9%)	
Legs	427 (47%)	257 (48%)	170 (45%)	
Feet	107 (12%)	60 (11%)	47 (12%)	
**Presence of FTB**	312 (40%)	164 (41%)	148 (37%)	0.415 ^
**In-patient status**	770 (95%)	376 (95%)	370 (97%)	0.149 ^
**Presence of inhalation injury**	117 (15%)	59(15%)	58 (16%)	0.812 ^
**Need for mechanical ventilation**	142 (16%)	86 (16%)	56 (15%)	0.360 ^
**In-hospital mortality**	72 (8%)	39 (7%)	33 (9%)	0.696 ^
Length of stay (days) *	23 (15-41)	24 (15-45.75)	21 (14-35)	0.014 ^#^

Control group = Burns admitted between 1 March and 31 December 2020; Study group = Burns admitted between 1 March and 31 December 2020; TBSA% = percentage of total body surface area burned; FTB = full thickness burn; BH = Belo Horizonte; LOS = Length of stay; * median (25th percentile, 75th percentile); # Wilcoxon Mann–Whitney Rank Sum test; ^ Chi Square test; ^†^ Fisher’s Exact test. Missing data for Age = 4, Place = 47, Cause = 6, Transferred = 48, Time to admission = 1, FTB = 14, In-patient = 12, Inhalation = 20, Mechanical ventilation = 16, Mortality = 8, LOS = 1.

**Table 2:** Differences between pediatric burn admissions before and during the COVID-19 pandemic.

	All Pediatric Burns (*n* = 277)	Control Group (*n* = 147)	Study Group (*n* = 130)	*p*
**Burn mechanism**				1 ^†^
Accident	271 (97%)	144 (97%)	127 (97%)	
Caused by other	4 (2%)	2 (2%)	2 (1%)	
Self-inflicted	2 (1%)	1 (1%)	1 (1%)	
**Burn cause**				0.10 ^†^
Scald	172 (62%)	92 (62%)	80 (62%)	
Flame	67 (24%)	36 (24%)	31 (24%)	
Chemical	4 (0%)	2 (0%)	2 (0%)	
Electrical	15 (0.5%)	8 (0.5%)	7 (0.5%)	
Contact	14 (0.5%)	8 (0.5%)	6 (0.5%)	
Other	3 (0%)	1 (0%)	2 (0%)	
**Place of injury**				0.16 ^†^
Outside	17 (1%)	10 (0.7%)	7 (0.7%)	
Work	2 (0.1%)	0 (0%)	2 (0%)	
School	3 (0.1%)	3 (0.2%)	0 (0%)	
Home	225 (81%)	111 (75%)	113 (86%)	
Other	17(1%)	11 (0.7%)	6 (0.7%)	

Control group = Burns admitted between 1 March and 31 December 2019. Study group = Burns admitted between 1st March and 31 December 2020. Data are numbers. † Fisher’s Exact test.
